# *XRCC1* gene polymorphisms and risk of neuroblastoma in Chinese children

**DOI:** 10.18632/aging.101601

**Published:** 2018-10-25

**Authors:** Jiao Zhang, Zhenjian Zhuo, Wenya Li, Jinhong Zhu, Jing He, Jinsong Su

**Affiliations:** 1 Department of Pediatric Surgery, the First Affiliated Hospital of Zhengzhou University, Zhengzhou 450052, Henan, China; 2 School of Chinese Medicine, Faculty of Medicine, The Chinese University of Hong Kong, Hong Kong 999077, China; 3 Department of Clinical Laboratory, Molecular Epidemiology Laboratory, Harbin Medical University Cancer Hospital, Harbin 150040, Heilongjiang, China; 4 Department of Pediatric Surgery, Guangzhou Institute of Pediatrics, Guangzhou Women and Children’s Medical Center, Guangzhou Medical University, Guangzhou 510623, Guangdong, China; 5 Department of Colorectal and Anal Surgery, The First Affiliated Hospital of Zhengzhou University, Zhengzhou 450052, Henan, China; * Equal contribution

**Keywords:** neuroblastoma, *XRCC1*, polymorphism, DNA repair, susceptibility

## Abstract

Neuroblastoma is a common pediatric extra-cranial tumor of the sympathetic nervous system. XRCC1 is a scaffold protein that participates in DNA single-strand break repair by complexing with other proteins. *XRCC1* gene polymorphisms are being increasingly explored in cancer epidemiology studies. However, the contribution of *XRCC1* gene polymorphisms to neuroblastoma risk remains unclarified. Herein, we conducted a case-control study with 393 neuroblastoma patients and 812 controls to explore the association of *XRCC1* gene polymorphisms (rs1799782 G>A, rs25487 C>T, rs25489 C>T and rs915927 T>C) with neuroblastoma risk. Results showed that none of the studied polymorphisms was associated with neuroblastoma risk. However, individuals with 2 risk genotypes seemed to be at significantly higher risk for neuroblastoma compared with those without risk genotype (adjusted odds ratio=1.69; 95% confidence interval=1.06-2.69). Stratified analysis revealed that the *XRCC1* rs25489 CT/TT was strongly associated with reduced risk of neuroblastoma in the children ≤ 18 months of age and subgroup with clinical stage I+II+4s diseases, compared with CC genotypes. We also identified an increased neuroblastoma risk for carrier of 2-3 risk genotypes among children ≤ 18 months of age and subgroup with clinical stage I+II+4s. More evidence of the association between *XRCC1* gene polymorphisms and neuroblastoma risk is needed.

## Introduction

Neuroblastoma is an extracranial solid tumor of the developing sympathetic nervous system [1,2]. This disease is the most common cancer in children under age of 1 year old, and accounts for about 7% of all childhood malignancies [2]. The prognosis of neuroblastoma varies greatly from spontaneous regression without chemotherapy to aggressive, incurable disease [3,4]. Approximately, 50% of affected children are diagnosed with a localized low- or intermediate- risk disease while rest of patients are of metastatic high-risk neuroblastomas [4–6]. The long-term survival rates of high-risk neuroblastoma cases remain disappointingly low despite of great advances in treatments [6–8].

The etiology of neuroblastoma is not fully elucidated [4]. Previous evidence has suggested that environmental risk factors for neuroblastoma, but no direct linkage is established in between [9,10]. Neuroblastoma is characterized by genomic instability and chromosomal abnormalities. Growing evidence indicated that the carcinogenesis of neuroblastoma is also partially attributed to the genetic factors and the gene-environment interactions. For instance, amplification of *MYCN* is observed in approximately 20% of neuroblastoma cases and associated with poor prognosis [11], and *ALK* mutation is identified in nearly 1% of neuroblastoma cases [12,13]. Recently, genome-wide association studies (GWASs) identified several neuroblastoma susceptibility loci [14–19]. Candidate gene approaches and fine mapping analysis also revealed that genetic variants in the *NEFL* [20], *CNKN1B* [21], and *BARD1* gene [22] are associated with neuroblastoma susceptibility.

*X-ray repair cross-complementing group 1* (*XRCC1*) was the first identified mammalian gene that is involved in the single strand DNA break repair [23]. XRCC1 serves as the scaffold protein without catalytic activity; alternatively, it facilitates single strand DNA break repair by cooperating with other proteins such as DNA polymerase β and DNA ligase III [24–26]. XRCC1 is mainly responsible for repairing DNA damage caused by ionizing radiation, alkylation, and UV exposure [27–29]. The abnormal function of XRCC1 protein could cause human cells more vulnerable to ionizing radiation and DNA-damaging agents. The associations between *XRCC1* polymorphisms and the risk of cancer have been widely investigated, including lung cancer [30], gastric cancer [31], and colorectal cancer [32]. However, no such association study has been performed in neuroblastoma. Give the significant role of XRCC1 in DNA repair, we conducted a case-control study to explore the association between *XRCC1* polymorphisms and risk of neuroblastoma.

## RESULTS

### *XRCC1* gene polymorphisms and neuroblastoma susceptibility

The characteristics of participants were described in our previous studies (Supplementary Table 1) [33–36]. The genotype frequencies of the four *XRCC1* polymorphisms (rs1799782 G>A, rs25487 C>T, rs25489 C>T and rs915927 T>C) in the neuroblastoma cases and controls are presented in Table 1. In the single locus analysis, none of the analyzed polymorphism was significantly associated with neuroblastoma risk. In analyzing the combined effect of risk genotypes, we found that subjects carrying 2 risk genotypes had a significantly increased neuroblastoma risk at an adjusted odds ratio (OR) of 1.69 [95% confidence interval (CI)=1.06-2.69] when compared with those carrying 0 risk genotype.

**Table 1:** Association between *XRCC1* gene polymorphisms and neuroblastoma susceptibility.

Genotype	Cases (N=393)	Controls (N=812)	*P* ^a^	Crude OR (95% CI)	*P*	Adjusted OR (95% CI) ^b^	*P* ^b^
rs1799782 G>A							
GG	182 (46.31)	406 (50.00)		1.00		1.00	
GA	177 (45.04)	333 (41.01)		1.19 (0.92-1.53)	0.186	1.19 (0.92-1.53)	0.187
AA	34 (8.65)	73 (8.99)		1.04 (0.67-1.62)	0.866	1.04 (0.67-1.62)	0.872
Additive			0.409	1.08 (0.90-1.30)	0.399	1.08 (0.90-1.30)	0.402
Dominant	211 (53.69)	406 (50.00)	0.230	1.16 (0.91-1.48)	0.230	1.16 (0.91-1.48)	0.231
Recessive	359 (91.35)	739 (91.01)	0.846	0.96 (0.63-1.47)	0.847	0.96 (0.63-1.47)	0.841
rs25487 C>T							
CC	221 (56.23)	471 (58.00)		1.00		1.00	
CT	151 (38.42)	278 (34.24)		1.16 (0.90-1.49)	0.260	1.16 (0.90-1.50)	0.257
TT	21 (5.34)	63 (7.76)		0.71 (0.42-1.19)	0.197	0.71 (0.42-1.20)	0.199
Additive			0.160	0.98 (0.81-1.19)	0.867	0.98 (0.81-1.20)	0.874
Dominant	172 (43.77)	341 (42.00)	0.560	1.08 (0.84-1.37)	0.560	1.08 (0.84-1.37)	0.554
Recessive	372 (94.66)	749 (92.24)	0.123	0.67 (0.40-1.12)	0.125	0.67 (0.40-1.12)	0.126
rs25489 C>T							
CC	326 (82.95)	636 (78.33)		1.00		1.00	
CT	64 (16.28)	170 (20.94)		0.73 (0.54-1.01)	0.056	0.73 (0.53-1.01)	0.055
TT	3 (0.76)	6 (0.74)		0.98 (0.24-3.93)	0.972	0.99 (0.25-4.01)	0.994
Additive			0.160	0.77 (0.57-1.03)	0.079	0.77 (0.57-1.03)	0.078
Dominant	67 (17.05)	176 (21.67)	0.061	0.74 (0.54-1.01)	0.061	0.74 (0.54-1.01)	0.060
Recessive	390 (99.24)	806 (99.26)	0.963	1.03 (0.26-4.15)	0.963	1.05 (0.26-4.24)	0.942
rs915927 T>C							
TT	294 (74.81)	627 (77.22)		1.00		1.00	
TC	97 (24.68)	169 (20.81)		1.22 (0.92-1.63)	0.165	1.23 (0.92-1.64)	0.158
CC	2 (0.51)	16 (1.97)		0.27 (0.06-1.17)	0.079	0.27 (0.06-1.16)	0.078
Additive			0.056	1.04 (0.81-1.35)	0.740	1.05 (0.81-1.35)	0.729
Dominant	99 (25.19)	185 (22.78)	0.356	1.14 (0.86-1.51)	0.356	1.15 (0.86-1.52)	0.345
Recessive	391 (99.49)	796 (98.03)	0.050	0.26 (0.06-1.11)	0.069	0.25 (0.06-1.11)	0.068
Combined effect of risk genotypes ^c^							
0	31 (7.89)	90 (11.08)		1.00		1.00	
1	240 (61.07)	509 (62.68)		1.37 (0.89-2.12)	0.158	1.38 (0.89-2.13)	0.150
2	121 (30.79)	210 (25.86)		**1.67 (1.05-2.66)**	**0.030**	**1.69 (1.06-2.69)**	**0.028**
3	1 (0.25)	3 (0.37)		0.97 (0.10-9.65)	0.978	0.96 (0.10-9.54)	0.969
Trend			0.154	**1.25 (1.02-1.53)**	**0.032**	**1.25 (1.02-1.54)**	**0.030**
0-1	271 (68.96)	599 (73.77)		1.00		1.00	
2-3	122 (31.04)	213 (26.23)	0.081	1.27 (0.97-1.65)	0.081	1.27 (0.97-1.65)	0.078

OR: odds ratio; CI: confidence interval. ^a^ *χ^2^* test for genotype distributions between neuroblastoma patients and controls. ^b^ Adjusted for age and gender. ^c^ The risk genotypes were rs1799782 GA/AA, rs25487 CT/TT, rs25489 TT and rs915927 TC/CC.

### Stratification analysis

We then further conducted stratification analysis for rs25489 C>T by age, gender, tumor sites of origin and clinical stages (Table 2). Compared to the rs25489 CC genotype, the protective effect of CT/TT genotypes was more predominant for children ≤ 18 months of age (adjusted OR=0.54; 95% CI=0.31-0.95) and those with clinical stage I+II+4s disease (adjusted OR=0.53, 95% CI=0.33-0.87). With the 0-1 risk genotypes as the reference group, the presence of 2-3 risk genotypes was associated with an elevated neuroblastoma risk among children ≤ 18 months of age (adjusted OR=1.87; 95% CI=1.19-2.93) and those with clinical stage I+II+4s tumor (adjusted OR=1.56, 95% CI=1.09-2.23).

**Table 2:** Stratification analysis of *XRCC1* rs25489 C>T polymorphism and combined risk genotypes with neuroblastoma susceptibility.

Variables	rs25489 (cases/controls)		OR (95% CI)	*P*	AOR(95% CI) ^a^	*P* ^a^	Combined (cases/controls)		OR (95% CI)	*P*	AOR (95% CI)^a^	*P* ^a^
	CC	CT/TT					0-1	2-3				
Age, month												
≤18	108/233 (27.48/28.69)	18/72 (4.58/8.87)	**0.54** **(0.31-0.95)**	**0.032**	**0.54** **(0.31-0.95)**	**0.032**	81/235	45/70	**1.87** **(1.19-2.93)**	**0.007**	**1.87** **(1.19-2.93)**	**0.007**
>18	218/403 (55.47/49.63)	49/104 (12.47/12.81)	0.87 (0.60-1.27)	0.473	0.87 (0.60-1.27)	0.469	190/364	77/143	1.03 (0.74-1.43)	0.852	1.03 (0.74-1.43)	0.851
Gender												
Females	141/262 (35.88/32.27)	27/80 (6.87/9.85)	0.63 (0.39-1.02)	0.058	0.63 (0.39-1.02)	0.058	115/252	53/90	1.29 (0.86-1.93)	0.217	1.28 (0.86-1.93)	0.227
Males	185/374 (47.07/46.06)	40/96 (10.18/11.82)	0.84 (0.56-1.27)	0.411	0.84 (0.56-1.26)	0.402	156/347	69/123	1.25 (0.88-1.77)	0.215	1.25 (0.88-1.78)	0.206
Sites of origin												
Adrenal gland	127/636 (32.31/78.32)	26/176 (6.62/21.67)	0.74 (0.47-1.17)	0.193	0.73 (0.47-1.16)	0.183	107/599	46/213	1.21 (0.83-1.77)	0.327	1.22 (0.83-1.78)	0.313
Retroperitoneal	74/636 (18.83/78.32)	13/176 (3.31/21.67)	0.64 (0.34-1.17)	0.146	0.64 (0.35-1.18)	0.151	60/599	27/213	1.27 (0.78-2.05)	0.337	1.27 (0.78-2.05)	0.332
Mediastinum	90/636 (22.90/78.32)	19/176 (4.83/21.67)	0.76 (0.45-1.29)	0.310	0.75 (0.45-1.27)	0.282	75/599	34/213	1.28 (0.83-1.97)	0.273	1.28 (0.83-1.98)	0.265
Others	27/636 (6.87/78.32)	9/176 (2.29/21.67)	1.21 (0.56-2.61)	0.637	1.21 (0.56-2.62)	0.636	23/599	13/213	1.59 (0.79-3.19)	0.193	1.60 (0.80-3.21)	0.189
Clinical stages												
I+II+4s	141/636 (35.88/78.32)	21/176 (5.34/21.67)	**0.54** **(0.33-0.88)**	**0.013**	**0.53** **(0.33-0.87)**	**0.011**	104/599	58/213	**1.57** **(1.10-2.24)**	**0.014**	**1.56** **(1.09-2.23)**	**0.015**
III+IV	168/636 (42.75/78.32)	43/176 (10.94/21.67)	0.93 (0.64-1.35)	0.683	0.93 (0.64-1.35)	0.698	154/599	57/213	1.04 (0.74-1.47)	0.817	1.04 (0.74-1.47)	0.823

AOR: adjusted odds ratio; CI: confidence interval. ^a^ Adjusted for age and gender in logistic regress models, without the corresponding stratify factor.

## DISCUSSION

In this two-center Chinese population study, we examined the impact of polymorphisms in the *XRCC1* gene on neuroblastoma susceptibility. We found that *XRCC1* polymorphisms might play a low-penetrant role on the neuroblastoma risk.

*XRCC1* gene is located on chromosome 19q13.2 and encodes a 633 amino acid protein [37]. It consists of 17 exons, spanning a genomic distance of 32 kb. By now, more than 8,453 polymorphisms in the *XRCC1* have been identified. Among them, three common polymorphisms, rs25487 C>T (Arg399Gln), rs25489 C>T (Arg280His), and rs1799782 G>A (Arg194Trp), are mostly investigated. These three polymorphisms could cause amino acid changes in evolutionarily conserved regions [23,38], and may further lead to the alteration in the XRCC1 protein activity. Previous study showed that *XRCC1* polymorphism 399Gln allele might affect the function of XRCC1 protein, consequently impairing DNA repair capacity [39].

Increasing epidemiological studies have been performed regarding the association between *XRCC1* polymorphisms and cancer risk. Hong et al. observed that rs25487 T allele and the 3 common allele combinations (rs25487 C>T, rs25489 C>T, and rs1799782 G>A) were associated with an increased risk of colorectal cancer in a study of 209 colorectal cancer cases and 209 controls recruited from Korean [40]. Divine et al. reported that *XRCC1* rs25487 TT genotype conferred susceptibility to lung adenocarcinoma at an OR of 2.8 (95% CI=1.2-7.9) in a case-control study [41]. In a population-based case-control study conducted among Korean population, Lee et al. found that none of the polymorphisms (rs25487 C>T, rs25489 C>T, and rs1799782 G>A) contributed to the risk of gastric cancer [42]. Duellet al. also reported a null association between *XRCC1* rs1799782 G>A and breast cancer risk in a population-based case-control study in North Carolina [43]. However, they observed a positive association for rs25487 CT or TT genotypes compared with CC among African Americans, but not Caucasians. This conflicting role of *XRCC1* polymorphisms indicates their risk effects may depend on cancer types and ethnic groups.

In the present study, we found that none of the four polymorphisms of *XRCC1* gene were associated with the risk of neuroblastoma. Failure to detect the association between *XRCC1* polymorphisms and neuroblastoma risk might be due to the small sample size and the low-penetrant effect of common polymorphisms. In the combined analysis, subjects carrying 2 risk genotypes tend to have increased neuroblastoma risk, when those without risk genotypes serve as a reference. Stratification analysis revealed that the rs25489 CT/TT is associated with favorable clinical markers such as ≤ 18 months of age and clinical stage I+II+4s. While exploring cumulative effects of risk genotypes, we found that the presence of 2-3 risk genotypes was associated with neuroblastoma risk among children ≤ 18 months of age and those with clinical stage I+II+4s tumors. It is believed that cancer susceptibility results from interactions of genetic and environmental factors and is subjected to modification of clinical markers such as age and clinical stage; in other words, the effect of one SNP on cancer susceptibility maybe modified by other genetic variants, environment exposures, and clinical makers. Single-locus analysis in the current study only focused on one genetic factor. the rs25489. Stratified analysis by age and clinical stage involve the effects of the rs25489 and clinical makers. While SNPs were combined, stratified analysis by age and clinical stage involve interaction of multiple SNPs and clinical makers. It is reasonable that analyses involved in different variables may lead to different results. Therefore, results considering interplays of gene-gene, gene-environmental factors and clinical makers maybe more meaningful in the clinical setting. Alternatively, these results could be chance findings, which calls for larger validation studies.

The study was limited by several points. Firstly, the relative small number of participants in the study impaired the statistical power. Therefore, the null association obtained in this study should be cautiously explained. Secondly, the insufficient information about environmental factors limited our ability to analyze gene-environment interactions. Thirdly, only four polymorphisms were investigated in the current study. More polymorphisms should be tested in the future work. Fourthly, due to the restriction to Chinese descent, these findings cannot be directly extrapolated to other ethnicities before validation studies. Last, our work lacks of *in vitro* data to support a role for these *XRCC1* polymorphisms in neuroblastoma, which should be completed in the future.

In conclusion, we report that polymorphisms in *XRCC1* gene may exert a low-penetrant effect on neuroblastoma risk in Chinese population. Our study helps to provide novel insight into the role of *XRCC1* gene polymorphisms in neuroblastoma risk. The findings should be further confirmed in other independent case-control studies.

## MATERIALS AND METHODS

### Study populations

Details of the selection criteria and characteristics of the study participants were provided elsewhere [33–36]. In brief, 393 neuroblastoma cases and 812 healthy controls were included in this study. The cases were from two medical centers, Guangzhou Women and Children’s Medical Center and the First Affiliated Hospital of Zhengzhou University. All the controls were selected from the same region as cases during the same period. Informed consent was obtained at the time of enrollment. This study was conducted under the approval of the Institutional Review Board of the participating hospitals.

### Polymorphism selection

Potentially functional polymorphisms in the *XRCC1* gene were selected using dbSNP database (http://www.ncbi.nlm.nih.gov/) and SNPinfo (http://snpinfo.niehs.nih.gov/) [44]. In final, four polymorphisms: rs1799782 G>A, rs25487 C>T, rs25489 C>T and rs915927 T>C, were adopted for analysis. As shown in our Supplementary Figure 1, there was no significant LD (R^2^<0.8) among these SNPs of *XRCC1* (R^2^=0.032 between rs25487 and rs25489, R^2^=0.039 between rs25487 and rs915927, R^2^=0.122 between rs25487 and rs1799782; R^2^=0.014 between rs25489 and rs915927, R^2^=0.043 between rs25489 and rs1799782, R^2^=0.046 between rs915927 and rs1799782).

### Genotyping

We used a TIANamp Blood DNA Kit (TianGen Biotech Co. Ltd., Beijing, China) for DNA extraction. Polymorphisms were genotyped applying TaqMan platform (Applied Biosystem, Foster City, CA, USA) without knowing the status of the participants [45–47]. Quality control was conducted by including eight negative controls with water in each plate. We also randomly selected 10% of the samples to perform a second genotyping, and the concordance rate was 100%.

### Statistical analysis

The Hardy-Weinberg equilibrium (HWE) was performed for each polymorphism among controls using the goodness-of-fit χ^2^ test. Two-sided chi-square test was applied to compare the demographic variables between the case group and controls. Tests for association between *XRCC1* polymorphisms and neuroblastoma risk were conducted by comparing the genotype frequencies between cases and controls using the χ^2^ test. We also applied stratified analyses by age, gender, tumor sites, and clinical stages. All analyses were conducted with SAS release 9.4 (SAS Institute, Cary, NC). *P*< 0.05 was defined as statistically significant.

## SUPPLEMENTARY MATERIAL

Supplementary Table 1

Supplementary Figure 1
