# Supplementary material for: XRCC1 gene polymorphisms and risk of neuroblastoma in Chinese children
Source: Aging (Albany NY). 2018 Oct 25;10(10):2944–53. doi: 10.18632/aging.101601 (PMC6224243; doi:10.18632/aging.101601)
Supplement: Supplementary Table 1 [file aging-10-101601-s001.pdf]

Supplementary Table 1. Frequency distribution of selected characteristics in neuroblastoma cases and cancer-free controls.

Variables	Guangdong province					Henan province				
	Cases (n=275)		Controls (n=531)		P [a]	Cases (n=118)		Controls (n=281)		P [a]
	No.	%	No.	%		No.	%	No.	%	
Age range, month	0-132		0.07-156		0.229	0-131.1		0.1-144.0		0.484
Mean ± SD	31.50±25.43		29.73±24.86			46.24±29.98		44.97±33.23		
<12	70	25.45	145	27.31		9	7.63	32	11.39	
12-60	177	64.36	313	58.95		76	64.41	179	63.70	
>60	28	10.18	73	13.75		33	27.97	70	24.91	
Gender					0.510					0.196
Female	114	41.45	233	43.88		54	45.76	109	38.79	
Male	161	58.55	298	56.12		64	54.24	172	61.21	
Clinical stages										
I	54	19.64				15	12.71			
II	62	22.55				31	26.27			
III	49	17.82				19	16.10			
IV	94	34.18				49	41.53			
4s	8	2.91				3	2.54			
NA	8	2.91				1	0.85			
Sites of origin										
Adrenal gland	64	23.27				89	75.42			
Retroperitoneal region	87	31.64				/	/			
Mediastinum	90	32.73				19	16.10			
Other region	26	9.45				10	8.47			
NA	8	2.91				/	/			

SD, standard deviation; NA, not available.

[a] Two-sided χ^2 test for distributions between neuroblastoma cases and cancer-free controls.
