# Supplementary material for: XRCC1 gene polymorphisms and risk of neuroblastoma in Chinese children
Source: Aging (Albany NY). 2018 Oct 25;10(10):2944–53. doi: 10.18632/aging.101601 (PMC6224243; doi:10.18632/aging.101601)
Supplement: Supplementary Figure 1 [file aging-10-101601-s002.pdf]

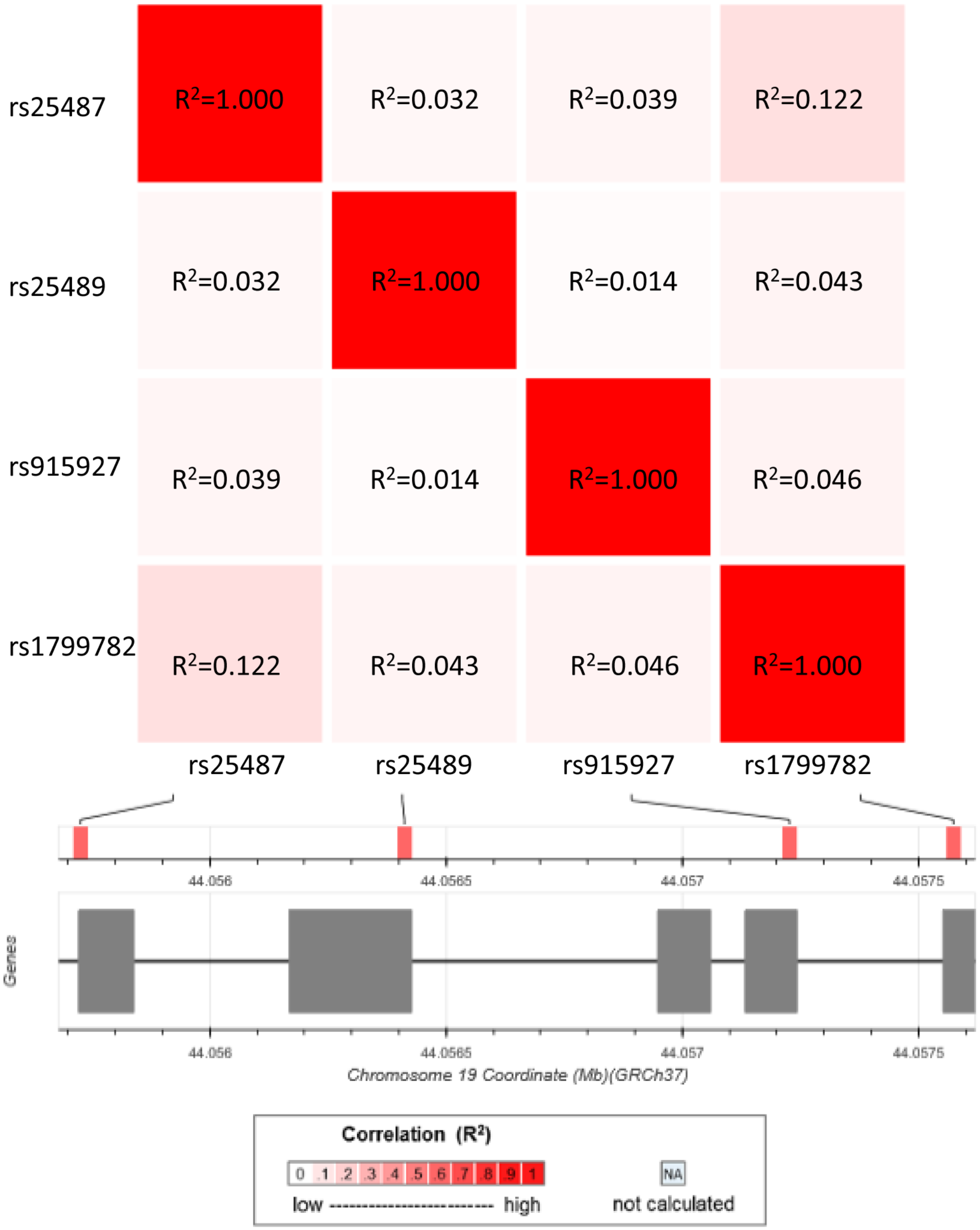

Supplementary Figure 1. Linkage disequilibrium analysis for the four selected *XRCC1* polymorphisms in Han Chinese population consisted of CHB (Han Chinese in Beijing, China) and CHS (Southern Han Chinese) subjects.
